# Supplementary figures and images for: Assessing future shifts in habitat suitability and connectivity to old-growth forests to support the conservation of the endangered giant noctule
Source: PeerJ. 2022 Nov 28;10:e14446. doi: 10.7717/peerj.14446 (PMC9744155; doi:10.7717/peerj.14446)

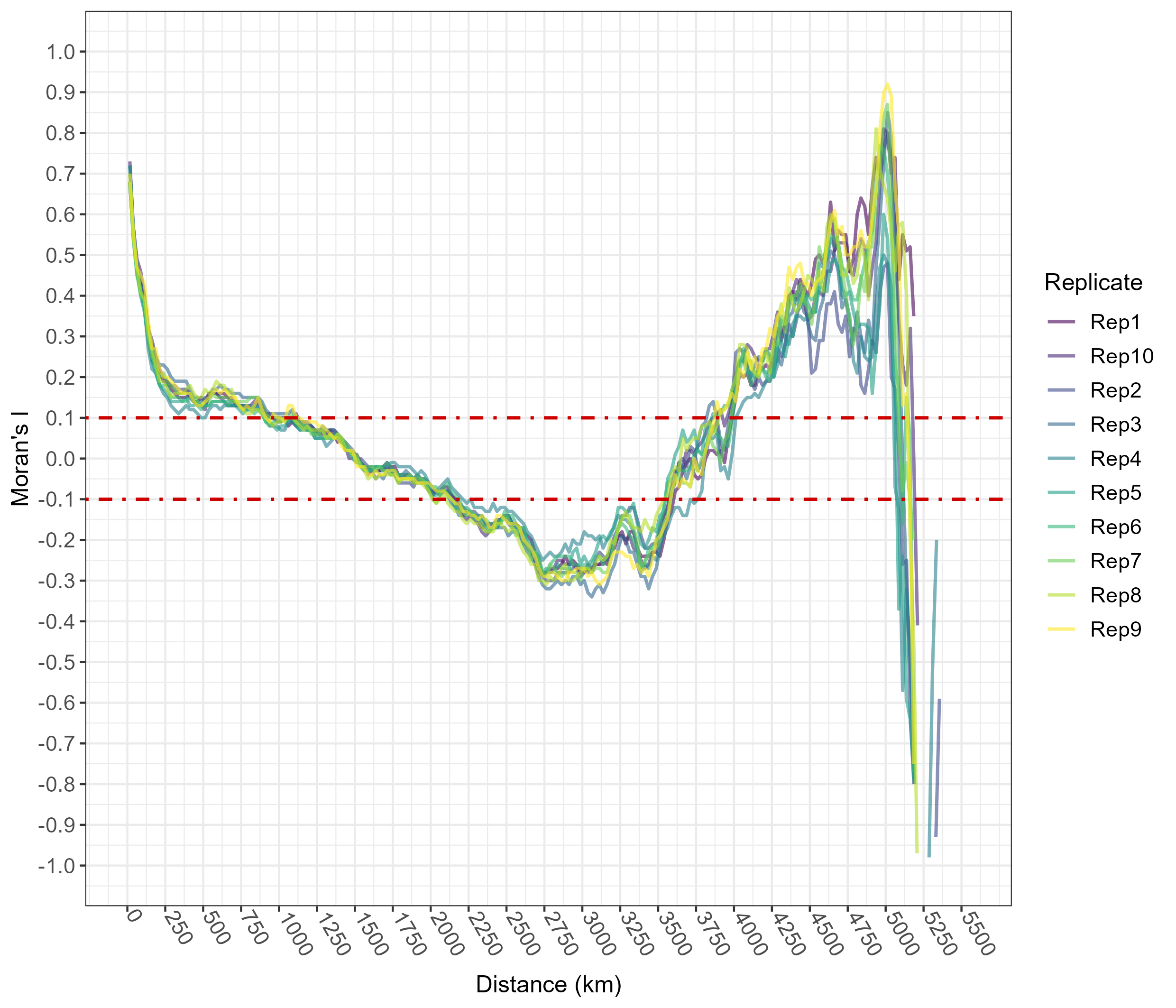

Supplement: Supplemental Information 4 — Correlogram showing variation of spatial autocorrelation, represented by Moran’s index (I), at increasing inter-point distances, within the residuals of 10 GBM models fitted using the optimized set of parameters selected through model tuning (see Materials and Methods – Ecological Niche Modelling in the main text). [file peerj-10-14446-s004.png]

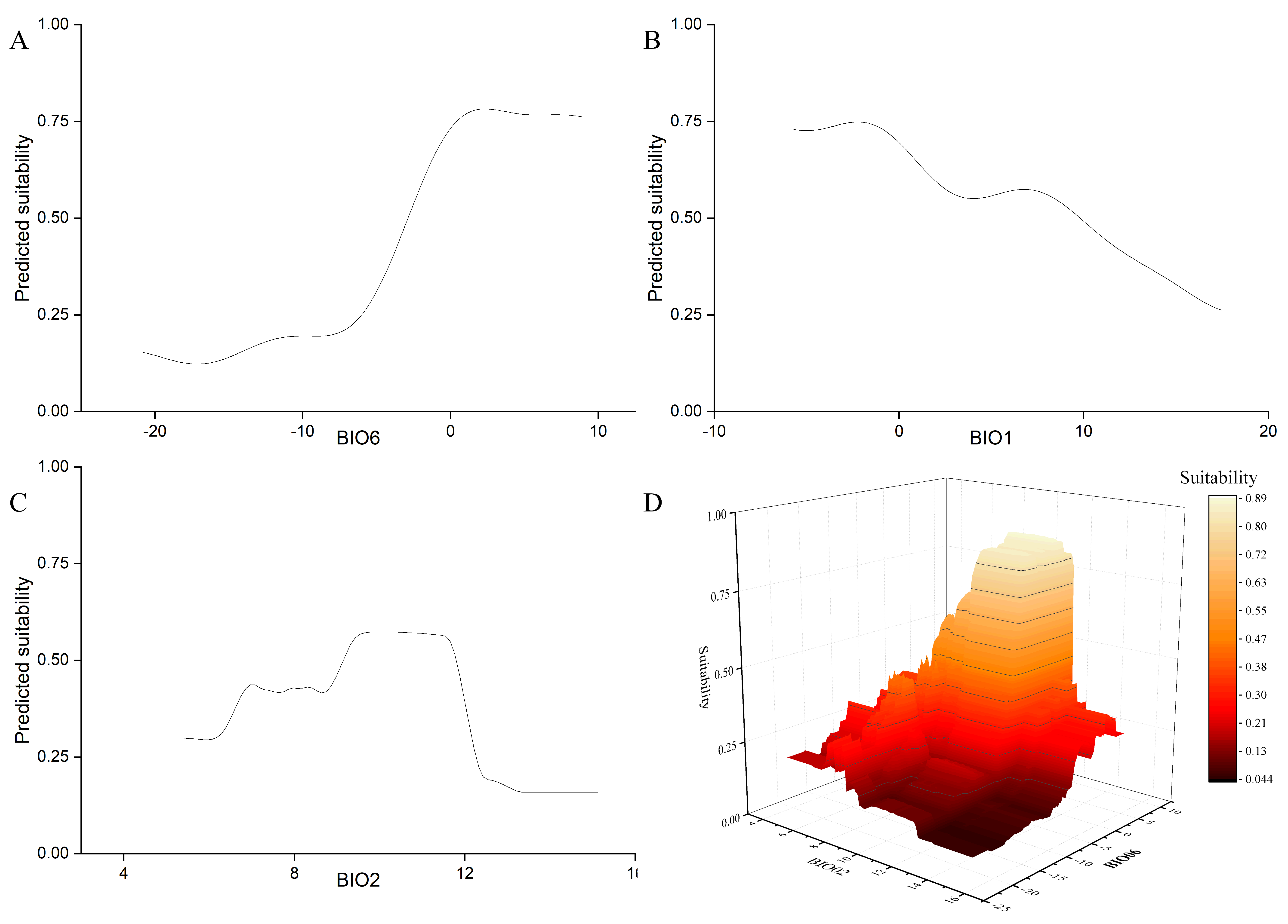

Supplement: Supplemental Information 5 — Marginal response curve, obtained from the optimized GBM model, for the three bioclimatic variables contributing the most to the modelled climatic suitability for Nyctalus lasiopterus under current climatic conditions: (A) BIO6 (percent contribution, 38.2%); (B) BIO1 (18.3%); (C) BIO2 (17.5%). (D) Marginal response surface showing the synergistic effect of BIO6 and BIO2 on predicted climatic suitability. [file peerj-10-14446-s005.png]

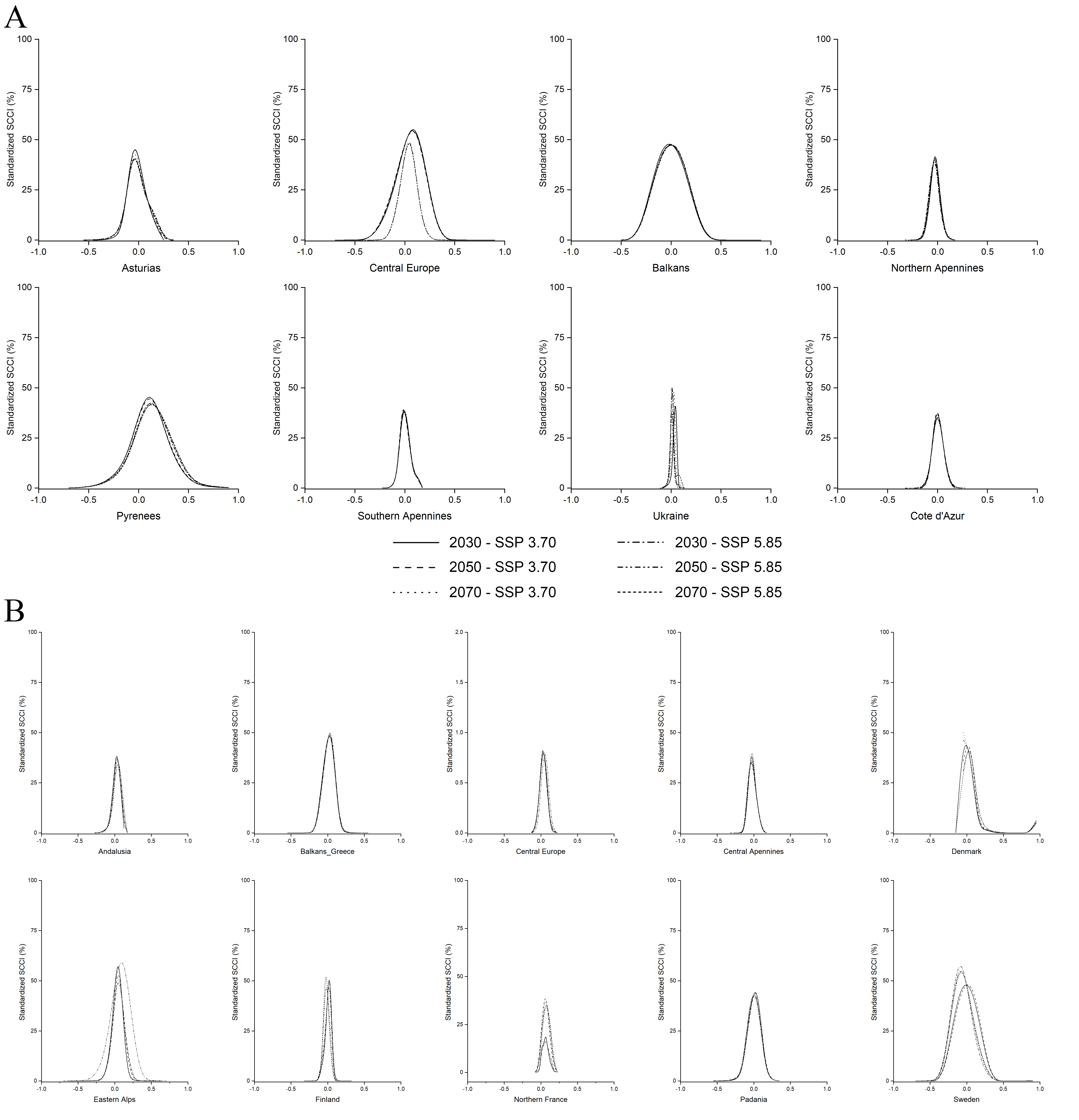

Supplement: Supplemental Information 6 — Distribution of Standardized Connectivity Change Index (SCCIs), between each of the considered future scenarios and current conditions, calculated for different European regions based on connectivity towards old-growth forests located within a 130-km-wide buffer around Nyctalus lasiopterus occurrence localities (A), or located outside the same buffer (B). [file peerj-10-14446-s006.png]
